# Short-Term Therapies for Treatment of Acute and Advanced Heart Failure—Why so Few Drugs Available in Clinical Use, Why Even Fewer in the Pipeline?

**DOI:** 10.3390/jcm8111834

**Published:** 2019-11-01

**Authors:** Piero Pollesello, Tuvia Ben Gal, Dominique Bettex, Vladimir Cerny, Josep Comin-Colet, Alexandr A. Eremenko, Dimitrios Farmakis, Francesco Fedele, Cândida Fonseca, Veli-Pekka Harjola, Antoine Herpain, Matthias Heringlake, Leo Heunks, Trygve Husebye, Visnja Ivancan, Kristjan Karason, Sundeep Kaul, Jacek Kubica, Alexandre Mebazaa, Henning Mølgaard, John Parissis, Alexander Parkhomenko, Pentti Põder, Gerhard Pölzl, Bojan Vrtovec, Mehmet B. Yilmaz, Zoltan Papp

**Affiliations:** 1 Critical Care, Orion Pharma, 02101 Espoo, Finland; 2 Heart Failure Unit, Rabin Medical Center, Tel Aviv University, Petah Tikva 4941492d, Israel; bengalt@clalit.org.il; 3 Institute of Anaesthesiology, University Hospital of Zurich, University of Zurich, 8091 Zurich, Switzerland; dominique.bettex@usz.ch; 4 Department of Anesthesiology, Perioperative Medicine and Intensive Care, Masaryk Hospital, J.E. Purkinje University, 400 96 Usti nad Labem, Czech Republic; vladimir.cerny@fnhk.cz; 5 Heart Diseases Institute, Hospital Universitari de Bellvitge, 08015 Barcelona, Spain; josepcomin@gmail.com; 6 Department of Cardiac Intensive Care, Petrovskii National Research Centre of Surgery, Sechenov University, 119146 Moscow, Russia; aeremenko54@mail.ru; 7 Department of Cardiology, Medical School, University of Cyprus, 1678 Nicosia, Cyprus; dimitrios_farmakis@yahoo.com; 8 Department of Cardiovascular, Respiratory, Nephrology, Anesthesiology and Geriatric Sciences, ‘La Sapienza’ University of Rome, 00185 Rome, Italy; Francesco.Fedele@uniroma1.it; 9 Heart Failure Clinic of S. Francisco Xavier Hospital, CHLO, 1449-005 Lisbon, Portugal; mcandidafonseca@gmail.com; 10 Emergency Medicine, Department of Emergency Medicine and Services, Helsinki University Hospital, University of Helsinki, 00014 Helsinki, Finland; Veli-Pekka.Harjola@hus.fi; 11 Department of Intensive Care, Experimental Laboratory of Intensive Care, Erasme Hospital, Université Libre de Bruxelles, 1050 Bruxelles, Belgium; Antoine.Herpain@erasme.ulb.ac.be; 12 Department of Anesthesiology and Intensive Care Medicine, University of Lübeck, 23562 Lübeck, Germany; Matthias.Heringlake@uksh.de; 13 Department of Intensive Care Medicine, Amsterdam UMC, Location VUmc 081 HV, The Netherlands; l.heunks@vumc.nl; 14 Department of Cardiology, Oslo University Hospital Ullevaal, 0372 Oslo, Norway; tr-huse@online.no; 15 Department of Anesthesiology, Reanimatology and Intensive Care, University Hospital Centre, 10000 Zagreb, Croatia; vivancan@kbc-zagreb.hr; 16 Transplant Institute, Sahlgrenska University Hospital, 413 45 Gothenburg, Sweden; kristjan.karason@medfak.gu.se; 17 Intensive Care Unit, National Health Service, Leeds LS2 9JT, UK; sunnykaul@aol.com; 18 Department of Cardiology and Internal Medicine, Nicolaus Copernicus University, 87-100 Torun, Poland; jkubica@cm.umk.pl; 19 Department of Anaesthesiology and Critical Care Medicine, AP-HP, Saint Louis and Lariboisière University Hospitals, Université de Paris and INSERM UMR-S 942-MASCOT, 75010 Paris, France; alexandre.mebazaa@aphp.fr; 20 Department of Cardiology, Århus University Hospital, 8200 Århus, Denmark; hennmoel@rm.dk; 21 Emergency Department, Attikon University Hospital, National and Kapodistrian University of Athens, 157 72 Athens, Greece; jparissis@yahoo.com; 22 Emergency Cardiology Department, National Scientific Center M.D. Strazhesko Institute of Cardiology, 02000 Kiev, Ukraine; aparkhomenko@yahoo.com; 23 Department of Cardiology, North Estonia Medical Center, 13419 Tallinn, Estonia; Pentti.Poder@regionaalhaigla.ee; 24 Department of Internal Medicine III, Cardiology and Angiology, Medical University of Innsbruck, 6020 Innsbruck, Austria; gerhard.poelzl@tirol-kliniken.at; 25 Advanced Heart Failure and Transplantation Center, Department of Cardiology, Ljubljana University Medical Center, SI-1000 Ljubljana, Slovenia; bojan.vrtovec@gmail.com; 26 Department of Cardiology, Dokuz Eylul University Faculty of Medicine, 35340 Izmir, Turkey; cardioceptor@gmail.com; 27 Division of Clinical Physiology, Department of Cardiology, Faculty of Medicine, University of Debrecen, H-4032 Debrecen, Hungary; pappz@med.unideb.hu; 28 HAS-UD Vascular Biology and Myocardial Pathophysiology Research Group, Hungarian Academy of Sciences, 4001 Debrecen, Hungary

**Keywords:** acute heart failure, advanced heart failure, short-term hemodynamic therapy, regulatory clinical trials, clinical development, levosimendan

## Abstract

Both acute and advanced heart failure are an increasing threat in term of survival, quality of life and socio-economical burdens. Paradoxically, the use of successful treatments for chronic heart failure can prolong life but—per definition—causes the rise in age of patients experiencing acute decompensations, since nothing at the moment helps avoiding an acute or final stage in the elderly population. To complicate the picture, acute heart failure syndromes are a collection of symptoms, signs and markers, with different aetiologies and different courses, also due to overlapping morbidities and to the plethora of chronic medications. The palette of cardio- and vasoactive drugs used in the hospitalization phase to stabilize the patient’s hemodynamic is scarce and even scarcer is the evidence for the agents commonly used in the practice (e.g., catecholamines). The pipeline in this field is poor and the clinical development chronically unsuccessful. Recent set backs in expected clinical trials for new agents in acute heart failure (AHF) (omecamtiv, serelaxine, ularitide) left a field desolately empty, where only few drugs have been approved for clinical use, for example, levosimendan and nesiritide. In this consensus opinion paper, experts from 26 European countries (Austria, Belgium, Croatia, Cyprus, Czech Republic, Denmark, Estonia, Finland, France, Germany, Greece, Hungary, Israel, Italy, The Netherlands, Norway, Poland, Portugal, Russia, Slovenia, Spain, Sweden, Switzerland, Turkey, U.K. and Ukraine) analyse the situation in details also by help of artificial intelligence applied to bibliographic searches, try to distil some lesson-learned to avoid that future projects would make the same mistakes as in the past and recommend how to lead a successful development project in this field in dire need of new agents.

## 1. Introduction

Despite the availability of successful treatments for chronic heart failure (CHF), acute heart failure (AHF) and advanced heart failure (AdHF) still impose considerable and rising health burdens in their impact on life expectancy and quality of life of an increasingly elderly population and the associated social and economic burdens.

AHF and AdHF syndromes are a collection of symptoms, signs and markers with different aetiologies, different clinical courses and different cardiac reserve. The innately complex nature of these conditions is further exacerbated by the fact that they are preponderantly encountered in an elderly population with overlapping morbidities and a plethora of chronic medications including drugs for serious co-morbidities.

In an acute episode, when a patient decompensates despite optimal p.o. medications, intravenous cardio- and vasoactive drugs are used to stabilize the situation. However, the repertoire of such drugs is relatively narrow—diuretics, vasodilators and inotropes—and evidence for sustained benefit of these agents is often strikingly thin—demonstration of mortality and morbidity gains in the long term remain elusive. Disappointingly, the number of successful innovations in recent years has been small. 

Recent experiences with an array of innovative cardio- and vasoactive drugs in acute heart failure were recently summarized in a review by Machaj et al. [1] (see Table 1 for studies on AHF). 

These results follow a course that has become familiar in this area of cardiovascular medical research in recent years—ingenious and scientifically plausible novel agents show often considerable promise in pre-clinical evaluations; that promise is carried forward into Phase I trials in humans and sometimes into Phase 2 trials in patients but pivotal or definitive Phase 3 trials interventions disappoint expectations and deliver no evidence of benefit on the nominated primary endpoint(s) or on clinically-relevant outcomes such as longer-term survival. Other authors offer similar tabulations and reach similar conclusions [2,3]. In advanced heart failure (AdHF) a recent update on the field had a conspicuous focus on developments in transplantation medicine and mechanical ventricular assist devices but was strikingly silent on the topic of medical innovations [4].

In a commentary published in 2014 reasons were identified that have contributed to this frustrating state of affairs [5]. Five years on and with the situation in many ways no better, it seems timely to re-visit this issue and to ask if the latest crop of negative clinical trials would trigger a reform of heart failure-targeted clinical research. We consider a root-and-branch reform to be essential if we are to break out of the pattern intimated in Table 1 and reinvigorate a therapeutic pipeline that with few exceptions has been painfully threadbare and unproductive for several decades. 

The main obstacles to progress already identified in that 2014 essay merit brief re-examination:

(1) The therapeutic field is complicated, the definitions of AHF and AdHF are not straightforward, with many aetiologies and various, often quickly evolving manifestations. Moreover, there is still a debate not only on the definition but also on the existence of some heart failure syndromes, for example, heart failure with mid-range ejection fraction [6] (HFmrEF). The combination of a broad-spectrum pathophysiology with vague definitions based on few parameters may preclude identifying meaningful group of patients benefitting of one particular drug instead of another. 

(2) The barrier to new entrants is set very high by the fact that regulatory clinical trials in AHF are targeted at demonstrating a reduction in longer-term mortality from drugs intended to be used as short-term interventions. Most trials are configured to evaluate the candidate drug as an addition to standard-of-care medications—that makes it very difficult to demonstrate significant and meaningful increases in survival (or indeed in lesser outcomes such as relief of dyspnoea) [7,8]. Hence, in order to deliver convincing findings, regulatory studies need to be both large and lengthy, leading to erosion of patent life. Additional complications relate to the regulatory requirements of populous emerging markets. The need to undertake both pivotal (often international) clinical trials but increasingly also locally-conducted single-country trials to secure marketing approval in some large national markets implies a duplication of funding and other resources, all of which add to the total costs of development and weaken the business case.

(3) The use of many traditional therapies with low levels of evidence to keep patients alive and to overcome the acute decompensation (e.g., generic intravenous vasodilators, diuretics, inotropes and vasopressors) makes it very difficult for new entrants to demonstrate a persuasive risk-benefit profile when the regulatory clinical studies must be conducted versus a placebo group that mandates use of extensive “standard of care” (SoC) therapy.

All these factors may discourage the sort of ambitious investment that might produce durable innovation and progress; other therapeutic areas may appear less risky or more rewarding to pharmaceutical companies considering where to place their research and development (R&D) effort. From a commercially-focussed perspective heart failure in all its manifestations, including AHF and AdHF, is a complex condition in which “there can be no realistic expectation of a blockbuster….and where it is wrong (both morally and commercially) to encourage hopes that such a drug is just around the corner [5].” As a corollary of this, the emergence of an era of personalized therapies implies both an opportunity and also an obligation to focus drug research in heart failure towards specific and precisely-defined sub-pathologies and to abandon, as irrational and futile, a search for a panacea. 

With no substantive additions to the therapeutic repertoire in recent times and the world of medicine (and indeed the world in general) poised for unprecedented changes in the nature, scale and accessibility of data, the argument for a complete revision of the theory, philosophy and practice of research in heart failure drug design is, we suggest, compelling. One early casualty of such a revision may be the end of almost any reliance on left ventricular ejection fraction (LVEF) as a primary metric in the characterization of heart failure.

## 2. A Systematic Analysis of the Past 20 Years

In order to understand better the field of drug development in the acute and advanced presentation of heart failure in the latest 20 years we performed an artificial intelligence (AI)-mediated search for all regulatory trials of Phase 3 on new chemical entities (NCE) aimed to validate the benefits of drugs developed for short-term treatment of AHF (including the wording “acutely decompensated heart failure”) and/or AdHF published after the year 2000. Clinical trials were searched using full-text search against studies’ descriptions with the NCE and the therapy area names. Using semantic similarity, studies implying semantic similarity of less than 30% with "heart failure" were filtered out. Reports exhibiting excessive semantic similarity with “kidney disease” or “addiction” were penalized to filter out studies focused principally on these topics and only mentioning heart diseases in passing (this condition applied for example, for studies involving dopamine). Comparator classes, first posting date of the study and patient enrolment were added to the data set. Finally, the results were checked independently by two researchers for their consistency. 

**Figure 1:**
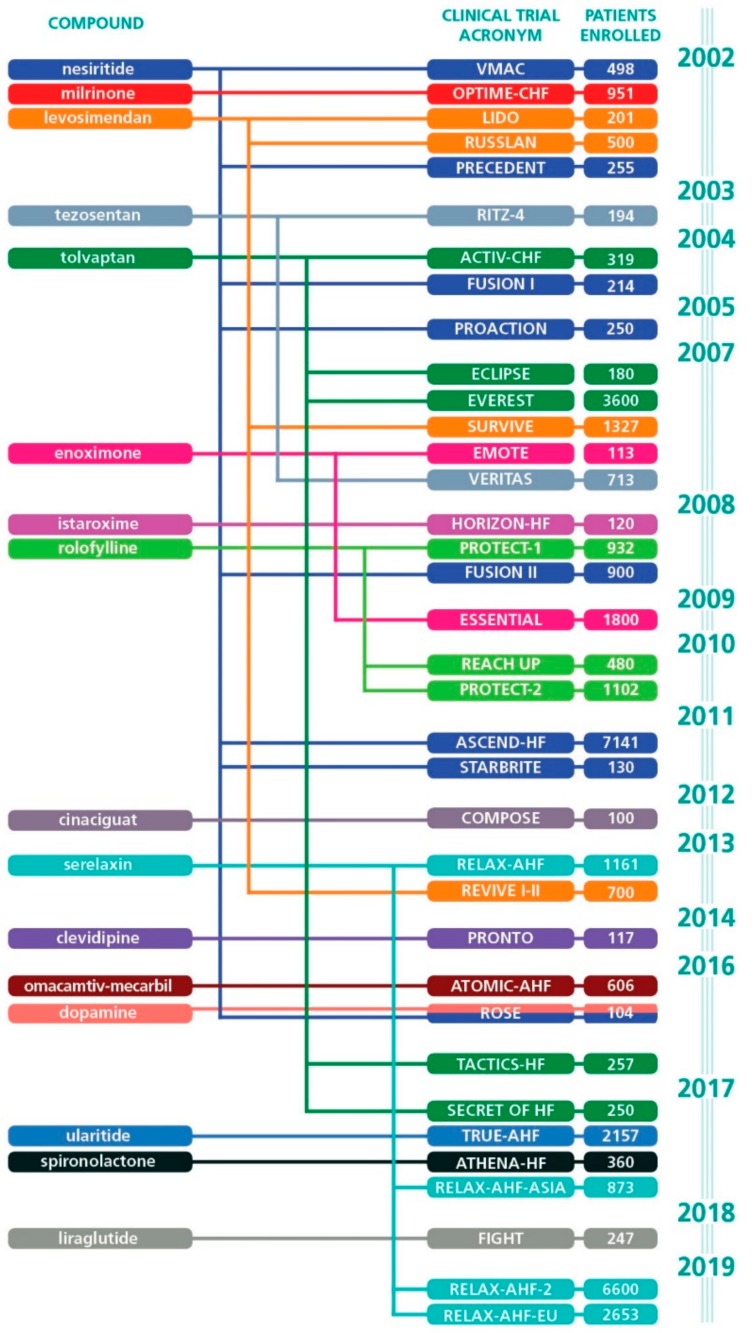
Regulatory clinical trials of Phase III for drugs meant for short-term treatment of acute heart failure (AHF) and/or advanced heart failure (AdHF), published in the past 20 years. For each study, the year of publication of the main report, the first author and the PMID are the following—VMAC, 2002, VMAC investigators, 11911755; OPTIME-CHF, 2002, Cuffe MS, 11911756; LIDO, 2002, Follath F, 12133653; RUSSLAN 2002 Moiseyev VS 12208222; PRECEDENT, 2002, Burger AJ, 12486437; RITZ-4, 2003, O’Connor CM, 12742280; ACTIV-CHF, 2004, M. Gheorghiade, 15113814; FUSION I, 2004, Yancy CW, 15342289; PROACTION, 2005, Peacock WF, 15915407; EVEREST, 2007, Konstam MA, 17384437; ECLIPSE, 2007, Udelison JE, published as abstract; SURVIVE, 2007, Mabazaa A, 17473298; EMOTE, 2007, Feldman AM, 17967591; VERITAS, 2007, McMurray JJ, 17986694; HORIZON-HF, 2008, Gheorghiade M, 18534276; PROTECT-1, 2008, Cotter G, 18926433; ESSENTIAL, 2009, Metra M, 19700774; FUSION II, 2008, Yancy CW, 19808265; REACH UP, 2010, Gottlieb SS, 20797594; PROTECT-2, 2010, Massie BM, 20925544; ASCEND-HF, 2011, O’Connor, 21732835; STARBRITE, 2011, Sha MR, 21807321; COMPOSE, 2012, Gheorghiade M, 22713287; RELAX-AHF, 2013, Teerlink JR, 23141816; REVIVE I-II, 2013, Packer M, 24621834; PRONTO, 2014, Peacock WF, 24655702; ATOMIC-AHF, 2016, Teerlink JR, 27012405; ROSE, 2016, Wan SH, 27512103; ROSE, 2016, Wan SH, 27512103; TACTICS-HF, 2016, Felker GM, 27654854; RELAX-AHF-ASIA, 2017, Sato N, 27825893; SECRET OF HF, 2017, Konstam MA, 28302292; TRUE-AHF, 2017, Packer M, 28402745; ATHENA-HF, 2017, Butler J, 28700781; FIGHT, 2018, Sharma A, 30120812; RELAX-AHF-EU, 2019, Maggioni AP, 30604559; RELAX-AHF-2, 2019, Metra M, 31433919.

**Figure 2:**
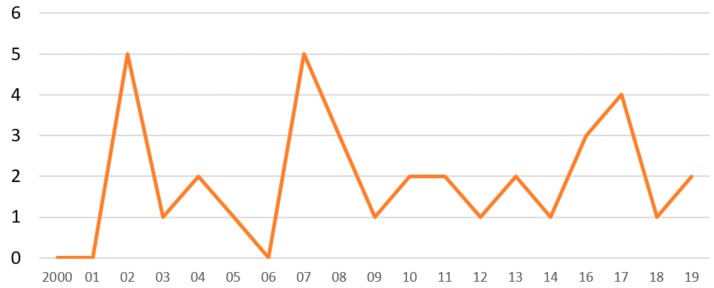
Amount of regulatory clinical trials of Phase III for drugs meant for short-term treatment of AHF and/or AdHF per year of publication in the period 2000–2019.

We identified 36 regulatory clinical trials in the past 20 years which were classified as Phase III (Figure 1). Those studies were aimed to test the hypothesis of clinical benefits of 16 different NCE (mainly exerting hemodynamic effects such as inotropy, vasodilation, diuresis), only few of which were finally approved in the U.S.A. or in Europe for use in AHF. By plotting the studies in chronological order, it can be seen that the density of Phase III trials publications has been consistently low in the past two decades (Figure 2). To be noticed is that, among the few trials in which the hypothesis was statistically proven, three (LIDO, RUSSLAN and REVIVE) tested the effect of levosimendan in AHF.

The total number of patients included in the 36 clinical trial was circa 38,000, notwithstanding that our search did not include either the Phase I and II trials or any Phase IV study. The fact that so many patients have been enrolled in this long series of inconclusive or negative studies should be considered of significance.

## 3. Also the Recent Clinical Trials have Disappointed

Before addressing these themes in more detail, it is appropriate to look briefly at some experiences in recent decades in the development of intravenous (i.v.) drug therapies.

Omecamtiv mecarbil binds with high affinity to the catalytic domain of myosin, increasing the number of myosin heads available to cross-link with actin. In theory, this augments cardiomyocyte contractility without increasing intracellular free ionic calcium or cardiomyocyte oxygen consumption [1]. In reality, the history of omecamtiv mecarbil might be seen rather as a demonstration that the pharma industry sometimes has a short memory. Candidate drugs which prolong the contractility transient were discontinued several decades ago because of their potential for harm in ischaemic conditions [9]. Omecamtiv mecarbil – at least at high plasma concentrations—shares some of these characteristics [10] and the ATOMIC-AHF trial produced a biomarker signal similar to that seen during myocardial infarction [11] (a higher median plasma troponin level) that might be related to cardiac ischemia described in an earlier Phase II trial [12]. The regulatory clinical programme for omecamtiv mecarbil in AHF has been halted. Insights on the safety and efficacy of oral omecamtiv mecarbil for chronic heart failure may be expected from the GALACTIC-HF study, due to complete in 2021. 

Serelaxin is a recombinant form of the endogenous hormone relaxin-2 and exerts vasodilatory, anti-inflammatory and anti-fibrotic effects [13]. The RELAX-AHF trial produced evidence of lower incidence of worsening heart failure during hospitalization [14] but the later RELAX-AHF-2 trial, which unlike RELAX-AHF, was powered for mortality, found no impact on 180-day cardiovascular mortality and a numerical but not statistically significant effect on worsening heart failure [15]. The challenge of deciding which of these sets of findings is most relevant to the treatment of AHF patients is evident. Further illustrations of the complexities and challenges of assigning weight to the results of clinical trials is provided by the demonstration that patients in RELAX-AHF were substantially unrepresentative of patients with AHF in the United States, Latin America or Asia-Pacific [16] and by the report that the RELAX-AHF-EU trial, yielded results similar to and supportive of RELAX-AHF [17] in the context of open-label drug administration.

Ularitide, a synthetic form of the human natriuretic peptide urodilatin, exerts vasodilator, diuretic and natriuretic effects via the natriuretic peptide receptor/particulate guanylate cyclase/cyclic guanosine monophosphate pathway and displayed beneficial effects such as symptom relief and vasodilation in animal models of heart failure as well as early-phase clinical studies in heart failure patients, In a Phase 3 trial (TRUE-AHF) in patients with acute heart failure, however, short-term ularitide treatment did not affect a clinical composite end point or reduce long-term cardiovascular mortality despite various nominally favourable physiological effects (and without affecting cardiac troponin levels) [18]. 

The early promise of istaroxime, which promotes the activity of sarco(endo)plasmic reticulum Ca(2^+^)-ATPase 2 (SERCA2) and thereby promotes expulsion of free intracellular ionic calcium through transmembrane sodium/calcium channels appears not to been sustained since the publication of the findings of the HORIZON study [19,20,21] and the results of the CUPID-HF study suggest that gene transfer of the SERCA2 gene is not yet a proven intervention [22]. 

Two studies of the nitroxyl (HNO) moiety (NCT01096043 and NCT10192325), commenced in 2010 appear to remain incomplete and unreported while evaluation of a follow-up molecule designated a BMS-986231 (previously CXL-1427) are in only preliminary stages [23]. The list of set-backs continues with tezosentan, nesiritide, tolvaptan, milrinone, enoximone, rolofylline, clevidipine, SLV320, cinaciguat, dopamine, liraglutide and high-dose spironolactone (see Figure 1).

## 4. Levosimendan—A Rare Case

Levosimendan, an inodilator that promotes contractility by binding to calcium saturated troponin C and vasodilatory and cardioprotective effects through the opening of adenosine triphosphate-dependent potassium (K_ATP_) channels is one of few agents of recent decades to establish itself in the medical repertoire for AHF and AdHF for its sustained hemodynamic, neurohormonal and symptomatic effects [24,25]. This status rests on findings from a series of Phase II and III studies published in the early 2000s. Two large post-approval clinical trials (SURVIVE and REVIVE) did not substantiate an indication of long-term effects but both a meta-analysis involving data from more than 6000 patients and a real-world registry involving over 5000 patients (ALARM-HF) were strongly indicative of long term survival benefit [26,27] – at a minimum to the extent that levosimendan use has never been associated with increased mortality, whereas the use of adrenergic/calcium mobilizing inotropes such as dobutamine has. At this regard, it is worth reminding that several authors in the past recognized a correlation between the effects of cardiovascular drugs on intracellular calcium and on long-term survival in heart failure, to the advantage of drugs which do not elevate either calcium transient or mitochondrial calcium, such as levosimendan [28,29,30]. 

Finally, since no attenuation of the hemodynamic effect of levosimendan is apparent in patients treated with beta-blockers [31,32] − now a substantial proportion of the overall heart failure population − the drug has been favoured for use in such patients in the most recent edition of ESC guidelines [33].

Levosimendan has also been evaluated in randomized controlled trials in advanced heart failure (Levo-Rep (NCT01065194), LION-Heart (NCT01536132) and LAICA (NCT00988806)) [34,35,36]. Observations in those trials are indicative of clinical benefits including reduction in NT-pro-BNP levels and trends towards reductions in heart failure readmissions and heart failure-related mortality. Those trends were corroborated in metanalyses where statistically significant reductions in long-term mortality and re-hospitalization were demonstrated [37,38] and subsequently in the RELEVANT-HF study, in which the addition of intermittent levosimendan therapy at 3-4 week intervals was associated over the course of 6 months with a substantially lower percentage of days in hospital (2.8 ± 6.6% vs. 9.4 ± 8.2%; *p* < 0.0001) and in the cumulative number and length of HF-related admissions (both *p* < 0.0001 vs. control), plus a marked but non-significant improvement in 1-year survival free from death/need for implantation of a ventricular assist device or urgent transplantation (86% vs. 78%) [39].

## 5. Even the Established Drugs May Not “Work.”

Very recently, at the European Society of Cardiology Congress, the GALACTIC trial reported that early intensive vasodilation using personalized high doses of nitrates, oral hydralazine and rapid up-titration of ACE inhibitors or angiotensin II receptor blockers did not improve 180-day mortality in a cohort of 781 acute heart failure patients [40]. This trial is notable, among other things, for the fact that short-term use of conventional “tried and tested” (and extremely cheap) vasodilators, administered in an intensive regime and at high dose was just as ineffectual at influencing longer-term mortality as novel agents such as ularitide and serelaxin. The inability to demonstrate survival benefit even from drugs that are established as part of the therapeutic armamentarium for AHF highlights some fundamental issues contributing to the paucity of new drug therapies in recent decades—for example, are we targeting the wrong pathological processes in our drug development programmes or are we privileging inappropriate endpoints in clinical trials and thus hampering the regulatory approval of useful new agents?

The general lack of evidence for an ongoing survival benefit from acute-phase treatments for AHF requires some reflection. While perhaps not fully subscribing to its philosophical outlook we find much to agree with in the views of McCullough [41], who has argued that AHF (and by extension AdHF) is a situation often long in the making and that to expect any therapy administered for ≤48 h to make a robust difference to survival or rehospitalization many months after the index admission is to misunderstand the pathophysiology of these conditions. 

## 6. Where Next and How to Get There?

Readers looking for a way forward from this seeming impasse may find encouragement in a recent review by Triposkiadis and colleagues [42]. We consider that publication to be a most significant contribution to this arena of cardiovascular research for the manner in which it articulates and crystallizes lines of critical thinking that have been apparent for some years but which, through advances in technology, are now poised to transform both the conception of heart failure and its modes of treatment.

A central premise of this work is that describing heart failure in terms of LVEF, while useful in its time, has become counter-productive and increasingly is obscuring the pathophysiological realities of heart failure, with adverse consequences for the evolution of therapy [43,44]. We concur with Triposkiadis et al. [42] that heart failure is “a heterogeneous syndrome in which functional and structural biomarkers change dynamically during disease progression in a patient-specific fashion” and that the condition as a whole may usefully be portrayed as a spectrum in which, depending on their proximity within that spectrum, individual presentations may or may not have overlapping phenotypes and shared underlying pathologies. Features of heart failure identified by Triposkiadis et al. [42] as occurring across the heart failure spectrum include:

1. Bidirectional transitions of LVEF due to disease treatment and progression
2. Endothelial dysfunction, cardiomyocyte dysfunction and cardiomyocyte injury
3. Systolic and diastolic left ventricular dysfunction
4. Left atrial dysfunction
5. Myocardial fibrosis
6. Skeletal myopathy
7. Heart failure serum markers
8. Neurohumoral activation

**Figure 3:**
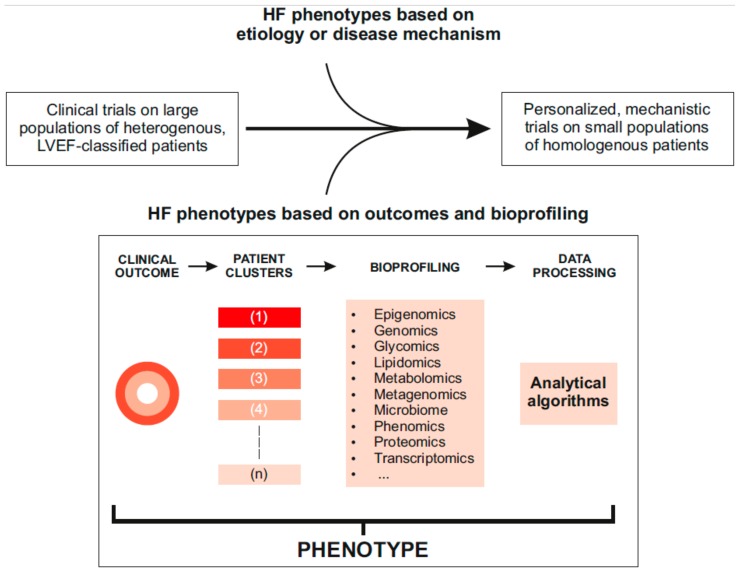
Advances in information and data-processing technology have created a base from which heart failure research can be re-configured towards highly defied phenotypes in ways that will facilitate both the optimal use of current therapies and the identification of new agents specifically tailored to a particular pathophysiology. See text for further discussion. Freely from Triposkiadis et al [42].

From that starting position Triposkiadis et al. [42] advocate the development of a wholly new classification of heart failure based on ultra-detailed phenotyping of the sort now made possible by advances in biological technologies and computing. This process, illustrated in Figure 3, proves a basis both for the better application of existing therapies and to shape the development of new agents. Two pathways of stratification are identified by this reasoning – one is hypothesis-driven, based for example on disease aetiology or mechanism or shaped by known pharmacological pathways of action; the other is hypothesis-free approach driven by the modern capacity to acquire unprecedented volumes of phenotype data and to analyse that data at unprecedented speeds and granularity, so identifying characteristics (or “signatures”) that differentiate sub-sets of patients with different heart failure phenotypes, different outcomes and different responses to various therapies.

Finally, in order to stratify HF patients, it should be mandatory to consider systematically the functions of other organs such as lung, kidney, liver, brain, hematopoietic system and so on, as proposed recently [45]. 

## 7. Invasive versus Non-Invasive Monitoring

When comparing the measures suggested for the diagnosis of AHF in the most recent European guidelines [33] with previous versions, an obvious trend to avoid invasive diagnostic measures (like using a pulmonary artery catheter) in AHF can be noticed. It would be wrong to rely primarily on simple clinical signs for assessing the severity and the type of failure in AHF—its complex manifestations and haemodynamic profile cannot be adequately diagnosed and differentiated by bedside assessment. Additionally, not any single word within 85 pages of the guidelines can be found on monitoring the systemic oxygen consumption and delivery by determination of mixed or at least central venous oxygen saturation; despite heart failure is classically defined as the inability of the heart, to maintain an adequate oxygen supply to the tissues. One may argue that the lack of progress in clinical development of new agents for treatment of AHF may be also explained by the inappropriate diagnostic measures and monitoring modalities recommended by the current guidelines and that even the best drugs will fail if they are inappropriately used within the multiple manifestations of heart failure. We should also consider alternatives to the Swan Ganz catheter, as recently reviewed by a large panel of experts [46]. 

## 8. Will the Data Revolution Break the Logjam?

These proposals may be seen in the wider context of an explosion in personal data accessible for analysis and the rapidly evolving science of AI. Timely recent reviews of these themes have been published, identifying both the opportunities and the many challenges that these new technologies present [47,48,49,50]. As non-experts in those fields we are constrained in what we might say with authority about these developments but concur with Sim [49] on several aspects of the use of mobile data-reporting devices in health, including the observations that ”Tracking and reporting data are a mean to an end not an end in itself” and that “Innovation in electronic sensing is in many ways outpacing the imagination for how these sensors can be used clinically.” The second of those sentiments may be seen a warning to expect some developments in data acquisition to turn out to be diversions (or blind alleys) in the clinical context. 

Beyond these thoughts is the much more significant challenge of sifting this unprecedented mass of data to identify signs, signals and biomarkers that are robust, reliable, meaningful and capable of being used to guide therapy. The work Deng and colleagues, who have advocated for pre-procedural gene expression profiles of peripheral blood mononuclear cells as indicative of longer-term survival prospects in patients with AdHF undergoing mechanical circulatory support, is an illustration of the immense and exciting potential in this area [51,52]. We are unreservedly positive for the longer-term prospects in this area but once more concur with the views of Sim [49] and others, about the challenges of successful implementation [53,54,55].

In the imminent era of Big Data as a day-to-day reality filtering the signal from the noise will be essential if clinicians are not to be simply overwhelmed by the volumes of information suddenly at their disposal. The USA alone is estimated to generate per annum 14 petabytes of data just from echocardiography results, a volume of material that defies exhaustive analysis by conventional methods [47]. Machine learning and AI may be central to the effective identification and analysis and orderly presentation of relevant data. Unsupervised machine learning (when computers are tasked to identify underlying relationships in a dataset) combined with ‘pan-omic’ analysis (i.e., genomics, proteomics, transcriptomics, metabolomics, etc.) from high throughput molecular profiling may provide a practical foundation for the sort of precision phenotyping aspired to by Triposkiadis et al. [42] and for ‘hyper-local analytics’ [47]. Deep learning, based on neural networks, is another aspect of the machine learning and AI revolution likely to find applications in cardiology [56,57]. 

The challenges of bringing machine learning and AI effectively into the practice of cardiology and more specifically into the management of heart failure are not to be underestimated (see Shameer et al. [47] and Johnson et al. [48] for excellent commentary on current methodologies and some of their pitfalls and limitations, including some observations on the cost barriers that may be encountered in acquiring biomedical data) but seem likely to be overcome within a short span of years. There are ample reasons for optimism in this area but confident prediction of what will become available and when and to what effect is beyond the powers of these authors. 

## 9. Trials Design—Time for a Change?

Research into new therapies for AHF and AdHF in recent decades has come to resemble the definition of insanity ascribed to Einstein—doing the same thing over and over again and hoping for a different result. One emerging therapy after another is added to the SoC repertoire in a Phase 3 trial and in that context each in turn fails to meet the prespecified endpoints for meaningful efficacy. 

We may have reached a stage where the broad-spectrum pathophysiology of HF, with different signs, symptoms and manifestations, different aetiologies and different patient co-morbidities, explored against a background of SoC medication, may preclude identifying meaningful incremental clinical benefits using traditional trial methodology. 

One response to this situation may lie in the adoption of a composite clinical endpoint evaluated in a hierarchical manner. The methodology ensures that all trial participants contribute to the overall outcome analysis through one or more of the specified outcomes; this has helpful practical implications for the number of patients needed and the length of follow-up required to generate endpoint data. Highly affirmative initial results have emerged from the ATTR-ACT study, which used this methodology to evaluate tafamidis in transthyretin amyloid cardiomyopathy [58] and the LeoDOR study (NCT03437226) is currently using a similar approach to outcome assessment in AdHF patients receiving intermittent cycles of levosimendan therapy [59,60]. 

More radical ways forward may include the adoption of Bayesian adaptive trial design, which facilitates the study of multiple treatment approaches and therapies in multiple patient phenotypes within a single trial, while maintaining a reasonable sample size [61]. Another possibility is the adoption of the group-sequential multi-arm multi-stage (MAMS) trial. The relative strengths and limitations of these methods have been reviewed in detail [62,63]. Overarching these methods is the concept of the “platform” trial, a clinical study with a single master protocol in which multiple treatments are evaluated simultaneously. This offers flexibilities such as dropping treatments for futility or adding new treatments during the course of a trial. Platform trials have the attraction of being able to deliver robust results with fewer patients and less time than a traditional two-arm trial [64]. 

Some cautionary comments are appropriate at this point. These emergent trial methods may be attractive for their statistical and methodological properties but their implementation in practice can be very highly resource-intensive, even by usual standards and especially when they include biomarkers. Essential preparation for Bayesian adaptive platform trials includes extensive stakeholder consultations, in-depth statistical modelling and definition of both the best outcome measures and intra-study endpoints. Morrell et al. [65], Hague and colleagues [66] and Schiavone et al. [67] have recently offered some observations on the practicalities of conducting platform trials, based on first-hand experience and enquiry. One aspect of note is that “the biomarker-stratified trial has the effect of making staff in the trial office aware of specific patients in a unique way compared to non-stratified trials,” despite anonymization” [65]. This represents a profound alteration to the human environment of clinical trials’ conduct, which may be amplified by the emergence of ‘decentralized’ clinical trials that are conducted via mobile health or telemedicine platforms and involve virtual recruitment, delivery of trial products direct to the participants’ homes and smartphone-assisted outcome assessment [49].

## 10. Some Views for the Future

The various trends and opportunities we have identified in this review outline a future for the development of treatments for acute or advanced heart failure perhaps very different from those of the past 20 years. Significantly, however, we might re-write that sentence to give an important different emphasis—*“The various trends and opportunities we have identified in this review outline a future for the treatment of acute or advanced heart failure perhaps very different from those of the past 20 years.”* Readers will note that this second description emphasizes changes in the usage of drugs over the development of new drugs. 

Our views in this regard are shaped by two notable recent publications [2,68], both of which have argued that what matters to patients who are hospitalized with a decompensation event is that they avoid further such hospitalizations and avoid the increase in mortality that occurs during the recovery phase. Viewed from that perspective the clinical stabilization achieved during the acute phase of hospital admission may be a secondary objective and to a substantial degree disconnected from the longer-term outcomes that patients prize. As Hamo and colleagues have pointed out [2], the physiological changes that lead to hospitalization take place days or weeks before hospitalization whereas the major adverse outcomes of death or rehospitalization mostly occur post-discharge. That temporal difference, combined with the now extensive evidence that acute-phase symptom relief with available therapies does not reliably improve long-term outcomes strongly suggests that either (a) relief of symptoms may be dissociated from central pathophysiological mechanisms; or (b) any pathophysiological pathway that is targeted by an acute-phase intervention is not going to be fully rectified by such short-term therapy. Conceivably both of these considerations may apply simultaneously.

Further, it is difficult to distinguish between patients suffering from the heart failure syndrome who still have cardiac reserve and respond to short-term therapy with stabilization and those who display a similar a clinical picture but show little benefit and poor outcome due to a totally worn out heart.

The need, as Hamo et al. [2] express it, to assign the right drug to the right patients at the right time to deliver meaningful benefit to AHF or AdHF patients is likely to be facilitated to a quite extraordinary degree by the developments in data acquisition and analysis we have acknowledged and the hyper-detailed phenotyping anticipated by Triposkiadis et al. [42]. That pathway of evolution in patient profiling might indeed provide insights and a firmer basis for the development of effective and successful new intravenous therapies. One possible outcome from this transformation is that the SoC repertoire that has dominated since the 1980s is finally superseded. The disappointing experience in clinical trials during the past 20 years of adding of new agents to SoC has created an impression that SoC rather than providing a foundation for further advances has acted as a glass ceiling through which newer agents struggle to break. With hyperdetailed insights into the pathophysiology of individual patients the way may finally be open to new agents precisely targeted to specific pathophysiological processes while the population-wide application of, say, ACE inhibitors and/or beta-blockers may come to be seen as too imprecise to be justifiable. 

It is not less plausible, however, that the data revolution might re-shape clinical strategy around AHF and AdHF into a very different course in which an incident of decompensation is seen as a cue to intensify and optimise out-patient management with the express purpose of preventing future re-hospitalizations. We note in this context recent encouraging results using machine learning algorithms for the early detection of acute cardiac decompensation [69,70] or estimation of a patient’s risk for early re-admission after an index event [71] plus descriptions of the use of machine learning and telemedicine to predict and monitor patients’ treatment adherence [49,72]. Similar technologies might also be deployed to optimise the functioning and performance of specialist AdHF units such as that recently described by Kreusser and colleagues [73]. The success of the TIM-HF2 (NCT01878630) trial of telemedical interventional management in reducing unplanned cardiovascular hospital admissions and all-cause mortality is also highly pertinent in this context [74,75].

In such a scenario the emphasis in the development of overall effective medical therapy for AHF and AdHF may well be towards drugs that can be accommodated in the outpatient repertoire (and therefore probably given orally) rather than towards drugs (probably given parenterally) that are intended for the management of a decompensation crisis. Repurposing of existing agents, including drugs with no current cardiology indication, guided by new in-depth knowledge of pathophysiology is another possible line of development [76]. An ultimate goal for such a pathway would be to develop patient monitoring to such a degree of immediacy and accuracy (“ecological momentary assessment [49]”) that decompensations are wholly avoided by prompt, appropriate clinical responses. Such a programme, if successfully implemented, might render the concept of “acute-phase intervention” substantially redundant by eliminating episodes of decompensation. A review of notable ongoing research in this area has recently appeared in this Journal [77].

As regards drug discovery and translational science in the field of acute cardiac care, the translational committee of the ESC-HFA issued some scientific bases [78] designed to pave the way towards the development of new agents but the preclinical field remains scarcely populated, with just some notable exceptions such as the calcium sensitizer/PDE inhibitor ORM-3819 [79,80].

## 11. Implications for Drug Development

Commercial and societal responses to this new world of ultra-detailed patient characterization and real-time monitoring must be considered. In an era of hyper-detailed patient profiling it may transpire that fitting the right drug to the right patient translates in practice to each new drug being appropriate for a small, even tiny, number of patients. “Heart failure” might be transformed into a myriad of orphan drug indications. The implications on the ‘evidence based medicine’ predilection for large trials is evident. The implications for commercial profitability and/or drug acquisition costs is even more prominent. 

Some pertinent and sharply framed observations that have recently emerged on the possibility of a not-for-profit model of antibiotic development might conceivably also come to apply to drug design in heart failure [81]. We take no position on whether such a shift would be inherently a good or bad thing but certainly it would mark a profound departure from the current model of drug discovery and development. (See Dungen et al. [82] for another perspective on this issue.) 

These financial pressures will not be confined to cardiology – medicine as a whole faces similar pressures and opportunities. To that extent we anticipate, therefore, that new arrangements for the funding of medical treatments will address these tensions—but we are not equipped to speculate about the form these new arrangements may take or any unintended consequences they may create.

## 12. Conclusions

Hospitalization for heart failure, whether as a presentation of AHF or a decompensation in the context of AdHF, results in a down-shift in the trajectory of the syndrome that is associated with worsening outcomes and patient quality of life and increased costs of care. Medical progress to address these challenges has substantially stalled in the past 20 years but advances in data technology and analytics, along with developments in clinical trials design now offer opportunities to re-envision heart failure as a complex pathophysiological continuum in ways that may help to bring a new generation of therapies into clinical use. Meanwhile it would be advisable for the clinicians to evaluate if the nearly total absence of evidence of benefit with some of the traditional i.v. drugs used in AHF and AdHF (such as the catecholamines or the phosphodiesterase inhibitors) warrants their elimination from routine use in favour of treatments where such evidence has been accrued (e.g., for levosimendan). 

## Figures and Tables

**Table 1:** Recent large-scale regulatory Phase III trials testing novel therapies for acute heart failure. Data extracted from Machaj et al. [1].

Agent Name	Omecamtiv Mecarbil	Ularitide	Serelaxin	
**Trial name**	ATOMIC-AHF	TRUE-AHF	RELAX-AHF	RELAX-AHF-2
**Registry number**	NCT01300013	NCT01661634	NCT00520806	NCT01870778
**Sample size**	614 AHF patients	2.157 AHF patients	1.161 pts hospitalized for AHF	6.600 AHF patients
**Outcomes**	• failed to meet the primary endpoint of dyspnoea improvement • increased SET	• no significant differences in primary endpoints • significant dyspnea reduction in 83% of eligible patients	• VAS AUC scale dyspnea improvement • fewer deaths at day 180	• failed to meet primary endpoints (180-day cardiovascular death and worsening heart failure through day-5)
**Observed adverse events**	• no difference in adverse effect rate compared to placebo	• adverse effect on dyspnea in 17% of ineligible patients (prohibited intravenous medications)	• infrequent hypotensive events	• no serious adverse events

Abbreviations: AHF, acute heart failure; VAS AUC, visual analogue scale area under the curve.
